# Trends and risk factors for suicide mortality in India from 2001–2019: National mortality study

**DOI:** 10.1371/journal.pgph.0005547

**Published:** 2026-01-09

**Authors:** Wilson Suraweera, Sonali Amarasekera, Lakshmi Vijayakumar, Shreelata Rao Seshadri, Patrick Brown, Ayanthi Karunarathne, Michael Eddleston, Prabhat Jha

**Affiliations:** 1 Centre for Global Health Research, Unity Health and Dalla Lana School of Public Health, University of Toronto, Toronto, Canada; 2 SNEHA, Chennai, India; 3 Public Health Foundation of India, Bangaluru, India; 4 Department of Statistics, University of Toronto, Toronto, Canada; 5 Centre for Pesticide Suicide Prevention, University of Edinburgh, Edinburgh, Scotland; 6 Nuffield Department of Population Health, University of Oxford, Oxford, England; McGill University, CANADA

## Abstract

One in four of all global suicide deaths occurs in India, yet the epidemiology of suicide mortality in India remains largely undocumented. We analyzed over 20,000 suicides among 829,000 deaths collected from 2001-2019 within a nationally representative random sample of about 1% of Indian homes using lay field reporting with dual central medical adjudication of causes. We applied suicide death proportions to national demographic totals to estimate death rates and used proportional mortality to examine risk factors. While suicide death rates fell by 1.5% annually, the absolute number of suicides remained constant at around 200,000 annually (or 3.8 million from 2001 to 2019) due to rising population. Over this period, 44% and 50% of all suicides occurred at ages 15–29 and 30–69 years, respectively. Suicide death rates declined fastest in women aged 15–29 years, particularly after 2015. Suicide death rates from poisoning, mostly organophosphate pesticides, fell but those from hanging rose. Suicide death risks at ages 15–69 years were about eight times higher in selected southern high-burden states than in selected northern states. Individual suicide risk was highest among sons or daughters’ in-law, rural residents, and men who drank alcohol. Suicide is preventable, but requires accelerating prevention policies to address acute social stress, ongoing efforts to reduce lethality of attempts and reliable epidemiological monitoring.

## Introduction

Global suicide death rates have fallen by about 2% a year from 2000 over the last two decades, but the World Health Organization (WHO) estimates that about 700,000 deaths from suicide occurred in 2021. Of these suicides, more than one in four occurred in India [1].

The age patterns of suicide in India involves high rates in younger adults aged 15–29 years, especially in women, which differs markedly from high-income countries [2]. The complex social pressures on women, including within marriage and extended family living arrangements, is a feature of Indian female suicides [3]. The social and economic fabric of India has changed substantially over the last two decades with illiteracy rates being halved, and per capita income rising about five-fold [4]. These social changes may also affect suicide rates. Suicide deaths are avertable with public health policies, mental health interventions, and by limiting access to toxic organophosphate pesticides [5].

Because of the importance of suicide, particularly in young adults, the United Nations (UN) adopted a one-third reduction in suicide death rates as its indicator for improved mental health in the Sustainable Development Goals. In high-income settings, progress in lowering suicide death rates and their distribution by geography, sex and by method of suicide, can be monitored using data from near-universal medical certification of deaths. By contrast, only 7 million (M) of the 10M deaths at all ages occurring annually in India are registered, and fewer than 2M have assigned a medically-certified cause [6]. Suicide and suicide attempts have been decriminalized but a requirement for mandatory reporting has hampered reliable data collection of suicide [7]. Suicide statistics rely on the National Crime Records Bureau (NCRB). However, the NCRB underestimates suicide death rates, particularly in younger and older women [2,8].

Since India represents such a large gap in global epidemiological evidence, here we analyze a nationally representative mortality study called the Million Death Study (MDS) conducted in collaboration with the Registrar General of India (RGI)’s Sample Registration System (SRS). The MDS involves dual, independent physician-coded verbal autopsy deaths generated from surveys every six months from 2001 to 2019. We document the trends in suicide mortality from 2001 to 2019 for each sex and by age, the geographic variation in these trends, the method of suicide, and key social and behavioral risk factors.

## Materials and methods

### Nationally representative mortality data sources

The details of MDS and SRS sampling design, study procedures and quality assurance are extensively documented [9–11] including an analyses of suicide deaths from 2001-3 using MDS data [2]. The SRS sample frame updates every ten years. The deaths were collected from over 3.6 million households surveying small sampling units (randomly selected villages or urban census blocks) within three distinct sample frames capturing a little under 1% of Indian homes: 6671 units from 1994-2003, 7597 units from 2004-13; and 8853 units from 2014-23). These were derived, respectively, from 1991, 2001 and 2011 population censuses. We had access to primary, individual data from 2001 to 2014. Data from 2015 onward are not publicly available. Therefore our analyses for 2015–2019 were conducted using aggregated data released by the Registrar General of India [12].

We also collated all possible chronological data related to suicides in India including annual statistics of suicides that are published by NCRB [13] and model-based suicide mortality estimates of the Global Burden of Disease (GBD) 2019 [14]. We examined the National Mental Health Survey (NMHS) 2015–16 [15] that surveyed suicidal risk including ideation, preparing and making a plan, repeated thoughts of suicide attempts and actual attempts among 34,800 individuals in 12 selected states (Punjab, Uttar Pradesh, Tamil Nadu, Kerala, Jharkhand, West Bengal, Rajasthan, Gujarat, Madhya Pradesh, Chhattisgarh, Assam and Manipur). These states covered 60% of India’s population.

### Classification of suicide deaths

We included all deaths that dual independent physician coders classified as intentional self-harm (International Classification of Diseases: ICD-10 code: X60-X84) [**16**] from the MDS for 2001–14. In addition, we reviewed centrally and re-assigned the deaths that one physician assigned any of X60-X84 code and other physician assigned any undetermined intent injuries (Y10-Y14, Y16-Y34, Y96-Y98) or unintentional injury deaths (V01-V99, W00-W99, X00-X44, X46-X52, X57-X59, Y40-Y86, Y88-Y89) of the cases originally coded as non-suicide deaths.

For deaths after 2014, we extracted grouped data from RGI published tables by age group (<15, 15–29, 30–44, 45–59, 60–69, 70 + years), rural, urban and richer or poorest states. Starting in 2015, the RGI transferred responsibility for physician coding to a local institution, retaining same dual anonymous physician coding and ICD-10 classifications. Field procedures remain unchanged. Thus, we expect quality of suicide deaths during 2001–14 to be consistent with those during 2015–19. Analyses of trends from 2001-14 and 2015–19 separately did not materially alter our results.

Historically, suicide mortality in India is more concentrated in five southern states: Tamil Nadu, Andhra Pradesh (divided in 2014 to form the new state of Telangana), Karnataka, and Kerala, constituting about two-fifths of all suicide deaths even though these states have only one-fifth of India’s population [**2**]. We refer to these five states as suicide high-burden states.

### Mortality estimates

As with previous MDS estimates [2,9,17,18], we applied the SRS inverse probability of selection as sampling weights to the suicide death proportions. We calculated suicide to all-cause proportions using three-year moving averages for each age and sex stratum for each state. We interpolated the mortality proportions, using standard statistical methods for missing death counts where applicable, as done previously [**10]**. We applied these proportions to the United Nations Population Division death totals (median variant) for India for each year [**19**], ensuring that state-level totals (based on relative death rates in the SRS) matched national totals for each sex and age-specific strata. We estimated death rates for 2001 and 2019 (beginning and end periods) by extrapolating the mortality spline curves. For comparisons across the years, we standardized death rates to the 2001 Indian census population. To obtain the mortality rates by methods of suicide we applied the sample-weighted proportions of various methods of suicide observed in the MDS to overall suicide rate for 2001–14. In this analysis, we were unable to separate adequately pesticides from non-pesticide poisoning, but other reports find that the majority of poisonings are due to toxic organophosphate pesticides [**5**]. We calculated the probability of suicide deaths at ages 15–69 years using the cumulative suicide death rates of 5-yearly age groups (see Table 1 footnote for formula) [**20**]. We estimated the annualized percentage change in mortality trends based on the slope of the best-fitted linear regression to each time series, with the intercept accounting for baseline variation.

**Table 1:** Study deaths from suicide, estimated national deaths and suicide death rates for India by sex and age group from 2001 to 2019.

Variable (and 95% CI)	Females	Males	Both sexes
Study deaths: suicide/ all deaths	8,106/ 356,746	12,021/ 472,596	20,127/ 829,342
Proportional suicide deaths	2.3% (1.8, 2.7)	2.5% (2.2, 2.9)	2.4% (2.1, 2.7)
National deaths (in thousands)	1,621 (1609,1633)	2,192 (2180, 2204)	3,813 (3790, 3835)
**Suicide death rate per 100,000 population**			
15–29 years	29 (18, 40)	23 (16, 30)	26 (17, 34)
30-69 years	14 (13, 15)	28 (23, 32)	21 (19, 24)
70 + years	16 (11, 21)	30 (21, 39)	22 (18, 27)
All ages	14 (10, 17)	17 (14, 21)	16 (12, 19)
Annual percent change (%)	-1.6 (-1.7, -1.5)	-1.5 (-1.5, -1.4)	-1.5 (-1.6, -1.5)
**Age 15–69 years**			
Death rate per 100,000	21 (9, 32)	26 (18, 34)	23 (14, 32)
National deaths in thousands	1,509 (1498, 1520)	2,055 (2047, 2063)	3,564 (3555, 3573)
Annual percent change	-1.7 (-1.8, -1.6)	-1.4 (-1.5, -1.3)	-1.5 (-1.6, -1.4)
Period risk of death (%)	1.0 (0.8, 1.2)	1.6 (1.5, 1.8)	1.2 (1.0, 1.4)

Notes: The detailed annual time series of suicide deaths for 2001–2019 are in S1 Table A-C in S1 Text. The percentage distribution of study deaths by age in years for groups <15, 15–29, 30–69 and 70 or more were for both sexes: 1.6%, 44.0%, 50.2%, 4.2%; for males: 1.2%, 35.7%, 58.6%, 4.5; for females: 2.2%, 56.4%, 37.6%, 3.8% respectively. Period risk represents the probability of death between ages 15–69 years at the observed death rates and in the absence of competing causes of death. Period risk = (1 − exp(−5 Σ rate_j_) where j = age group in 5-year age groups. Average annual percent change is calculated using the linear trend. 95% confidence intervals (Lower, Upper) are shown in parentheses. We applied a best-fit linear regression model, to calculate the APC. We tested alternative modeling approaches, including Poisson and log-linear models, and found the resulting APC estimates to be highly consistent.

### Geospatial and risk factor analysis

We derived the spatially-smoothed absolute risks of suicide mortality in India for 2005–13 (ignoring 2004 as its results were less stable in the first year of the new SRS sampling frame). The methods used have been detailed previously [**10**]. Briefly, this involves death and population counts at ages 15–69 by 5-year age groups and sampling units. The model includes smooth year and age effects, a continuously varying spatial random effect, and an independent sampling unit effect. We applied a Generalized Linear Geostatistical model [**21**] to infer the model parameters (including the spatial range and variance) and spatial relative risk. Spatial relative risks were multiplied by the 15–69 year period risk derived from the age-specific rates, to estimate the corresponding absolute risk.

We calculated age-adjusted Mantel-Haenszel odds ratios (OR) to evaluate the risk linked to the following exposures adjusted for time period: being a resident in a high-burden state, rural residence, smoking or alcohol use, and the family relationship of the deceased person to the head of household, suicide deaths were cases and non-suicide deaths were controls.

## Results

From 2001 to 2019, the MDS-SRS recorded 829,300 all causes deaths of which 20,127 (female 8,106, male 12,021) were suicides. The overall proportional suicide deaths from 2001-19 was 2.4% (female 2.3%; male 2.5%; Table 1). Applying these proportions to national death totals yielded 3.81M suicides (95%CI 3.79, 3.83) over the 19-year period. Female totals were 1.62M [1.61, 1.63] or 43% and male totals were 2.19M [2.18, 2.20] or 57%. Suicides at ages 15–29, 30–69, and 15–69 years, were about 1.64M (44%), 1.92M (50%) and 3.56M (94%), respectively of all suicides. The highest death rates for both sexes were at ages 15–29 years with an average risk of suicide death between 15 and 69 years of 1.2% (female 1.0% [0.8, 1.2]; male 1.4% [1.2, 1.7]). Annual deaths risks between ages 15–69 years varied from 0.8% to 1.6% (S1 Table A-C in S1 Text). From 2001 to 2014, about 83% of suicides occurred in rural areas, and 62% at home, with the greater proportions of home suicide deaths among females, above age 70 years, and from hangings (S1 Table D in S1 Text).

**Fig 1:**
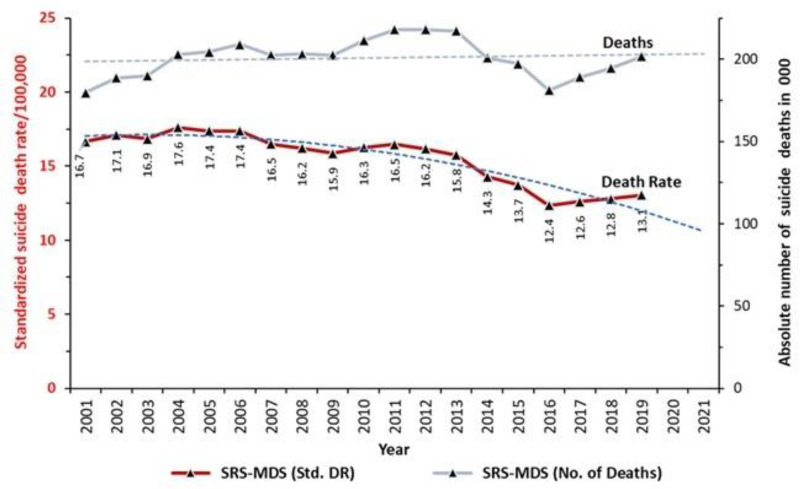
Suicide mortality trends in India, 2001–2019, based on SRS-MDS estimates. Rates standardized to the 2001 Indian census population. Estimated suicide deaths in thousands (right sided axes).

Age-standardized death rates from suicide fell over the 19-year period (Fig 1) at an annual rate of 1.5% (females 1.6%; males 1.5%). With population growth, however, the absolute number of annual suicide deaths remained about 200,000 throughout the period.

Data from the NCRB which uses police reports showed far lower suicide death rates, but converged gradually with MDS death rates, with the gap shrinking from 41% in 2001–3–29% in 2017–19 (S1 Table E in S1 Text).

**Fig 2:**
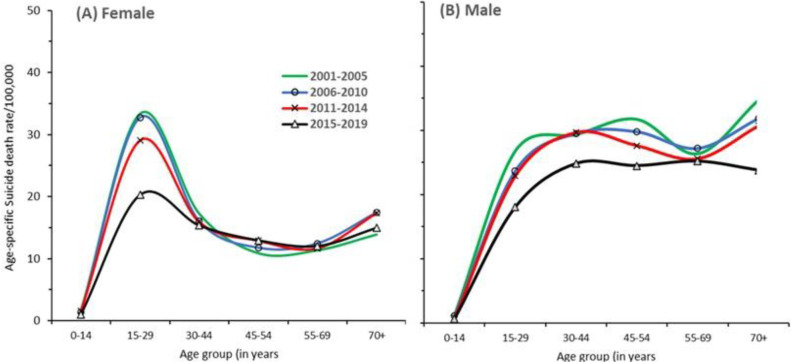
Age-sex patterns of suicide death rates from 2001-2019 study period. (A) Female, (B) Male. Each curve represents age-specific periodic death rates calculated using annual deaths and averaged within four time periods 2001-5, 2006-10, 2011-14 and 2015-19.

Suicide mortality declined fastest at ages 15–29 years with annual rates of decline being 2.4% in females and 2.0% in males (Fig 2, S1 Table B-C in S1 Text). Much of the reduction in female and male suicide rates appeared between the last (2015–19) and penultimate (2011–14) periods. For females at ages 15–29, suicide death rates fell by nearly by one third between these two periods. For males, declines of about one fifth were seen both at ages 15–29 and 30–44 years. The NCRB data also showed the fastest declines in younger adults (using their published age groups of 30–44 years; S1 Fig B in S1 Text). From 2001 to 2014, using the individual level MDS data, similar declines were seen in the five high-burden states and in the rest of India, even though the peak suicide death rates in both sexes were much higher in the high-burden states. We further explored if the sharp decline in suicide mortality, particularly at 15–29 years, could have arisen from causes potentially misclassified with suicide. Murder death rates fell in both sexes. Motor vehicle accidents rose, as expected. Ill-defined injuries rose only during 2015–19. Collectively, these patterns do not appear to suggest substantial misclassification of suicide deaths with other injury deaths (S1 Table F in S1 Text).

**Fig 3:**
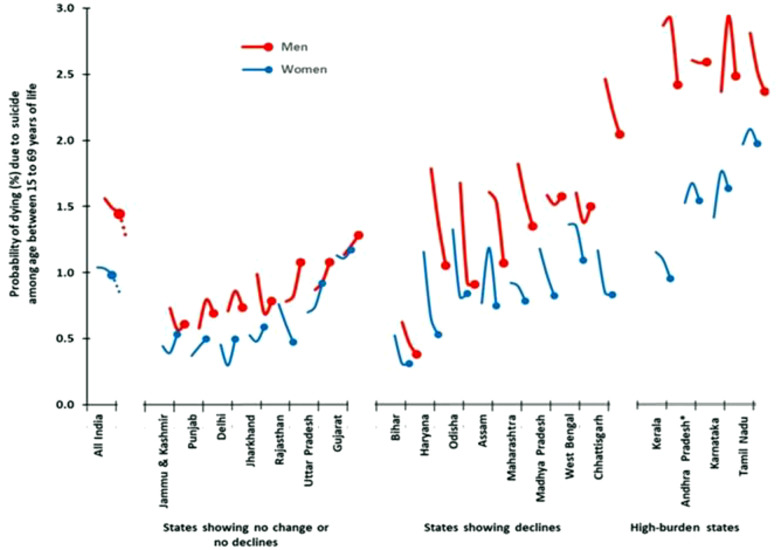
Trends in probability of dying from suicide among individuals aged 15 to 69 years in the larger states of India.

There was marked geographic variation in suicide death rates per 100,000 population (S1 Table G in S1 Text) with the high-burden states showing female and male rates of 23.9 and 31.0, respectively in 2011–14. These were approximately twice the suicide death rates for other states, but the individual state level contrasts were even sharper. For example, among men, death rates in Karnataka (33.9) were about eight times higher than in Bihar (4.2). There was also marked variation by region and state in the changes in the risk of suicide death between ages 15–69 years between 2001 and 2014. The overall risk for males fell from 1.7% to 1.4% and from 1.0% to 0.8% for females. Most states showed declines in both sexes, including all the high-burden states. However, several states, including Jammu and Kashmir, Punjab, Delhi, Jharkhand, Rajasthan, Uttar Pradesh and Gujarat showed no change or no decline in suicide morality risks (Fig 3; S1 Table G S1 Text). There was a marked excess of male over female suicide mortality risks in Chhattisgarh and Kerala, despite declining mortality risks fell in both states. The state level NCRB data from 2001 to 2019 showed similar decline patterns to the MDS trends from 2001 to 2014 with strong correlations between NCRB and MDS data trends for females and males (S1 Fig B-C in S1 Text).

**Fig 4:**
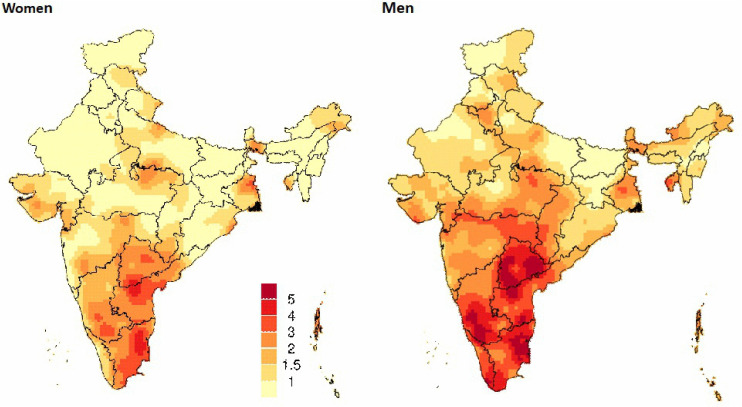
The spatial distribution of suicide mortality risk among men and women aged 15 to 69 years in India 2005-2013. Maps show the spatial mortality risk by suicide for individuals’ age between 15 to 69 years. Mortality risk was calculated using data collected from about 7500 randomly selected small sampling areas (SRS sample units) from 2005 to 2013 annual survey rounds. A Generalized Linear Geostatistical model was used [21]. State boundaries are from gdma.org [22]. We focused on the age 15–69 year group, which accounts for over 95% of suicide deaths, during which misclassification with other causes is less likely that at older ages. Larger states are those with a population > 10 million. Andhra Pradesh includes Telangana. Each curve illustrates the annual trend in probability of dying from suicide (expressed as a percentage) among individuals aged 15–69 years from 2001–2014. The dotted extension segment of the “All India” curve represents the national-level trend from 2015 to 2019 period, for which only national level data were available. The states are arranged in ascending order of overall suicide mortality risk (for both sexes combined) in 2014. States are further grouped based on the overall patterns of their suicide mortality trends over time. High-burden states are those where the age-standardized suicide death rate exceeds 20 per 100,000 population. Additional details are in S1 Table G in S1 Text.

The spatial distribution of suicide mortality risks generated from smoothed death rates across ~7500 sampling units for 2005–2013 (Fig 4) showed particularly high suicide death risks at ages 15–69 years among men in the high-burden states for both men and women. More focal excesses of death risks were seen in parts of Andhra Pradesh and Telangana for men and women (albeit at lower risk levels than in men).

**Fig 5:**
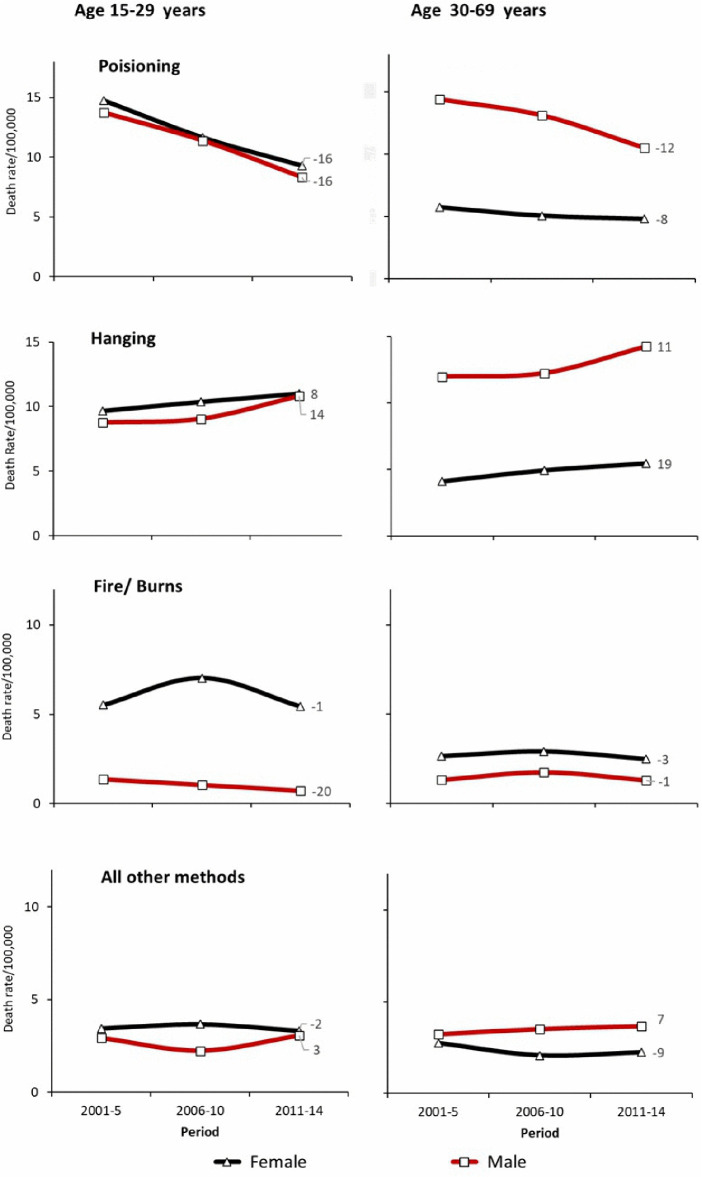
Trends in known method of suicide deaths by age and sex from 2001 to 2014. 3 digits ICD 10 codes: Hanging (X70), Poisoning including pesticides (X60-X69), Fire/Burns (X75-X76), All other methods (X71-X74, X77-X84). Trends are for 2001–2014. The label value shown at the end of each curve represents the relevant average annual percent change in mortality rates from 2001 to 2014.

Based on the ICD-10 codes, we distinguished suicide deaths as poisoning (combining X68 for pesticides and X60-X67, X69 for other poisonings), hanging (X70), fire/burns (X75-X76), and all other methods (X71-X74, X77-X84). From 2001-05–2011–14, hanging death rates rose in nearly at both ages 15–29 and 30–69 years in both sexes at an annual rate of 11% in each sex at ages 15–69 years. By contrast, poisoning showed declines in both age groups and each sex, at an annual rate of 14% in men and 12% in women at ages 15–69 years. The specific contribution of organophosphate pesticides to this decline could not be determined, as the data did not adequately differentiate pesticide types involved in poisoning deaths. Fire and burns were far more prevalent in women, and peaking in the youngest women aged 15–29 years by 2006–10 followed by a subsequent decline (Fig 5).

The NMHS of 2015–16 [**15**] has already reported an overall prevalence of suicidal ideation or risk of about 6% in 12 states and a prevalence of moderate or high risk of suicide in the last month of 0.72% (0.71-0.74) and 0.90% (0.89-0.92), respectively. Although the NMHS covered the diversity of India's regions, its suicide risk based on symptoms showed a poor geographic correlation with the prevalence of suicide death rates in the MDS or NCRB data (S1 Fig C in S1 Text).

**Fig 6:**
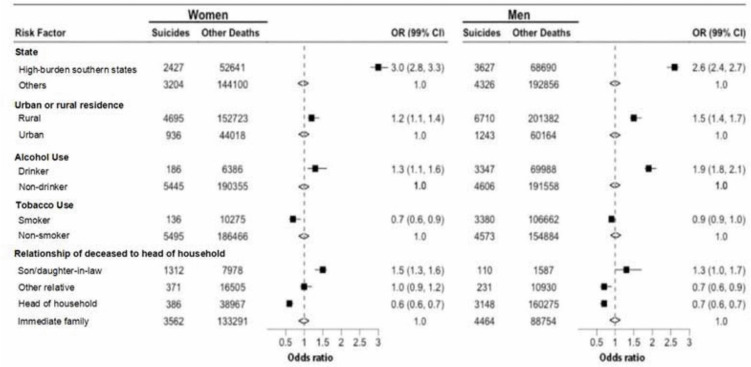
Selected risk factors associated with suicide deaths by sex, based on 2001 to 2014 study data. Mantel-Hanzal odds ratios and the Wald’s 99% confidence intervals represent odds ratio (OR) of suicide deaths over all other causes of death at exposure to each risk factor. Each risk factor adjusted to age and all other risk factors. The underlying data for this analysis were from 499,283 verbal autopsy causes of deaths of age over 15 years recorded from 2001 to 2014. Reference groups used in the analysis are as follows; other states for state of residence; urban for residence; non-drinker for alcohol use; non-smoker for smoking; and immediate family (spouse, son/daughter, brother/sister, grandparents or grandchildren living in the same household) for relationship to head of households. High-burden states are Andhra Pradesh, Telangana, Karnataka, Kerala and Tamil Nadu where the standardized suicide death rate exceeds 20 per 100,000 population.

Proportional mortality analyses comparing individual suicides to all other deaths, and adjusted for every other variable, showed that living in a high-burden state was associated with about a three-fold higher odds ratio of suicide deaths (Fig 6). Increased odds ratios (OR) were observed for rural residence, male alcohol drinkers but not for smoking in either sex. Being a son or daughter-in-law who lived with the parents in same household was independently associated with suicide in both women and men. Notably, the association of rurality, being a son or daughter-in-law and drinking alcohol appeared to be proportionately stronger in the high-burden states than in other Indian states (S1 Fig D in S1 Text).

## Discussion

Suicide in India continues to be a substantial and largely avertable burden, accounting for about 200,000 deaths a year. Suicide mortality is concentrated at working ages of 15–69 years and in particular at ages 15–29 years. The declines in overall suicide mortality in India of about 1.5% per year from 2001-2019 are slower than the global declines of about 2% per year [23,24]. The sharpest declines observed were younger women aged 15–29, particularly after 2015, during a time of a major expansion of economic growth and literacy. Reasons for these declines at these ages remain unknown. Possible explanations include may reflect the indirect effects of changing family dynamics, broader social and policy advances, including improved access to female education, increased labor force participation, targeted social welfare and empowerment programs, expanding digital connectivity that enhanced mental health awareness and support networks, and banning of acutely toxic organophosphate pesticides.

The age patterns and the lack of clear geographic correlation with suicidal ideation suggest that underlying chronic poor mental health, including major depression, may be less predictive than social factors, which are more challenging to measure in surveys [25,26]. We observed the highest risk of suicide mortality among rural residents, male alcohol drinkers and among sons and daughters in-law, and particularly so in the high-burden states in South India. There were marked gender gaps, not only with higher suicide death rates at ages 15–29 years among females than males, but also in markedly higher risk of suicide at age 15–69 years in men than in women in the very diverse states of Kerala (with high levels of education, and female labor force participation) and in Chhattisgarh (with large tribal populations). The marked regional differences in suicide mortality must reflect the existence of some underlying social, behavioral or biological including genetic risk factors that await further discovery.

Public health action to prevent suicide and reduce lethality attempts is feasible. This includes acute social crisis support for women and men facing social or work pressures that lead them to communicate distress, anger, powerlessness through self-harm. This behavior is distinct from purposeful actions to end their life [**25**]. Various non-governmental organizations have established crises lines and other acute services including online support, but these are yet to be scaled to national coverage. While our study does not show a strong ecologic link with mental health comorbidities, these conditions, particularly major depression, remain important. Thus, strengthening access to prevention, counselling and mental health treatment is warranted. In parallel, the integration of artificial intelligence and machine learning into health surveillance systems offers opportunities to enhance reliable epidemiological monitoring, refine trend forecasting, and enable proactive identification of mental health risks to guide public health action [**27**].

Efforts to restrict access to toxic organophosphates which began in Kerala in 2005 and regulatory actions by other states after 2011 [**28**] are likely to reduce suicide deaths. We observed declines in poisoning deaths preceding actual restrictions in toxic pesticides, which may be a result of greater awareness of pesticide-induced suicide in India. Pesticide bans are particularly effective at reducing deaths in young women, who self-poison at high rates compare to other ages and males [**29**]. Restrictions on toxic pesticides have reduced suicide deaths in Sri Lanka and other countries [**30**], and the federal government and states are adopting restrictions [**31**].

Hangings rose, consistent with the idea that suicidal ideation remains common in many parts of India.^15^ The geographic clustering of suicides within states, for example being prominent in southern Chhattisgarh in tribal areas, might also enable use of local data to scale up prevention and treatments for suicide attempts. Although we did not directly examine farmer suicide deaths, which have received widespread publicity, the 2001–19 trends remain consistent with our earlier analyses of 2001–3 deaths that found male farmer deaths to be a minority of overall suicides. Earlier analyses of MDS data suggested that extremes in water availability, either exceptionally wet or dry conditions, predict suicide mortality [**32**].

Our study strengths and some limitations. We use consistent nationally representative sampling frames with a robust number of suicides. Our results are broadly consistent with the modelled-based trends in suicide from the GBD for 1990–2016, but we avoid the inherent problems of modelling complex social phenomena using limited data [**33**]. Moreover, the GBD relies extensively on the NCRB which, despite recent improvements, continues to under-estimate suicide death rates. As far as we can determine, no other study has examined long-term trends (10 + years) in suicide mortality amongst Indians using reasonably contemporary and representative data. Our major limitation is potential misclassification of causes of death from verbal autopsies [**9**]. However, our analyses of possible competing categories of causes did not suggest that misclassification accounted for the falling suicide death rates, especially at ages 15–29 years. While suicide is reasonably easy to classify as a cause of death on verbal autopsies, stigma and social pressures may lead to under-reporting, particularly among younger women [**2**]. The continuous nature of the SRS field work, and the fact that the SRS surveyors are well known to the people in the sampled villages and urban blocks over many years likely mitigated but did not eliminate such under-reporting.

Some of the data uncertainties about recent years, including the effects of COVID-19, could be addressed if the RGI would enable open access to the primary data from 2015 and onwards and released its mortality data for 2020–21 and beyond. Such access would allow for a more comprehensive assessment of suicide trends and strengthen the temporal analyses presented in Fig 3–6.

India’s National Suicide Prevention Strategy aims to reduce the suicide mortality by 10% by 2030 [**34**], but this is too modest a goal. Building on the WHO’s Comprehensive Mental Health Action Plan of 2013–30, a more robust goal would be to reduce suicide by one-third by 2030 compared to 2015 rates [**35**]. Further progress in avoiding suicide deaths will require ongoing public debate and attention to what often remains a stigmatized topic, ongoing efforts to prevent suicide and to decrease lethality of various modes of intentional self-harm (in particular pesticide poisoning), and robust, reliable epidemiological and monitoring studies. Given that India constitutes a quarter of global suicides, reductions in suicide mortality in India could reduce the global burden of suicide deaths.

## Supporting information

S1 Text Supplementary web appendix. Table A in S1 Text – Annual suicide study deaths, estimated deaths and death rates for India from 2001 to 2019 – both sexes combined. Table B in S1 Text – Annual suicide study deaths, estimated deaths and death rates for India from 2001 to 2019 – for Females. Table C in S1 Text – Annual suicide study deaths, estimated deaths and death rates for India from 2001 to 2019 – for Males. Table D in S1 Text – Percent suicide deaths at home 2001–2014. Table E in S1 Text- Annual suicide deaths (in thousands) and death rates per 100,000: Comparison between current study and NCRB data in India, 2001–2019. Table F in S1 Text- Annualized proportional suicide deaths and other mis-classifiable injury, and ill-defined causes of deaths during study periods 2001–5, 2006–10, 2011–14 and 2015–19. Table G in S1 Text – Suicide mortality trends from 2001 to 2014 in larger states of India. Fig A in S1 Text - Age-sex patterns of suicide deaths observed in NCRB (All-India) and in the present study across High-burden and Other states. Fig B in S1 Text - State-wise comparison of suicide mortality trends for 2001–2019 between the present study (MDS) and the NCRB. Fig C in S1 Text – Correlation between prevalence of suicide morbidity risk in states surveyed by National Mental Health Survey (NMHS) 2016 and suicide mortality rates from the present study and NCRB data. Fig D in S1 Text - Age-adjusted combined risk factor effects associated with suicide vs other causes of deaths in suicide high-burden states and other Indian states.
(DOCX)
