# Supplementary material for: Trends and risk factors for suicide mortality in India from 2001–2019: National mortality study
Source: PLOS Glob Public Health. 2026 Jan 9;6(1):e0005547. doi: 10.1371/journal.pgph.0005547 (PMC12788641; doi:10.1371/journal.pgph.0005547)
Supplement: S1 Text — Table A in S1 Text – Annual suicide study deaths, estimated deaths and death rates for India from 2001 to 2019 – both sexes combined. Table B in S1 Text – Annual suicide study deaths, estimated deaths and death rates for India from 2001 to 2019 – for Females. Table C in S1 Text – Annual suicide study deaths, estimated deaths and death rates for India from 2001 to 2019 – for Males. Table D in S1 Text – Percent suicide deaths at home 2001–2014. Table E in S1 Text- Annual suicide deaths (in thousands) and death rates per 100,000: Comparison between current study and NCRB data in India, 2001–2019. Table F in S1 Text- Annualized proportional suicide deaths and other mis-classifiable injury, and ill-defined causes of deaths during study periods 2001–5, 2006–10, 2011–14 and 2015–19. Table G in S1 Text – Suicide mortality trends from 2001 to 2014 in larger states of India. Fig A in S1 Text - Age-sex patterns of suicide deaths observed in NCRB (All-India) and in the present study across High-burden and Other states. Fig B in S1 Text - State-wise comparison of suicide mortality trends for 2001–2019 between the present study (MDS) and the NCRB. Fig C in S1 Text – Correlation between prevalence of suicide morbidity risk in states surveyed by National Mental Health Survey (NMHS) 2016 and suicide mortality rates from the present study and NCRB data. Fig D in S1 Text - Age-adjusted combined risk factor effects associated with suicide vs other causes of deaths in suicide high-burden states and other Indian states. (DOCX) [file pgph.0005547.s001.docx]

# S1 Text. Supplementary web appendix

**Trends and Risk Factors for Suicide Mortality in India from 2001-2019: National Mortality Study**

**Appendix: Tables**

Table A – Annual suicide study deaths, estimated deaths and death rates for India from 2001 to 2019 – both sexes combined

Table B – Annual suicide study deaths, estimated deaths and death rates for India from 2001 to 2019 – for Females

Table C – Annual suicide study deaths, estimated deaths and death rates for India from 2001 to 2019 – for Males

Table D – Percent suicide deaths at home 2001-2014

Table E - Annual suicide Deaths (in thousands) and death rates per 100,000: Comparison between current study and NCRB data in India, 2001–2019

Table F - Annualized proportional suicide deaths and other mis-classifiable injury, and ill-defined causes of deaths during study periods 2001-5, 2006-10, 2011-14 and 2015-19

Table G – Suicide mortality trends from 2001 to 2014 in larger states of India

**Appendix Figures**

Fig A - Age-sex patterns of suicide deaths observed in NCRB (All-India) and in the present study across High-burden and Other states

Fig B - State-wise comparison of suicide mortality trends for 2001–2019 between the present study (MDS) and the NCRB

Fig C– Correlation between prevalence of suicide morbidity risk in states surveyed by National Mental Health Survey (NMHS) 2016 and suicide mortality rates from the present study and NCRB data

Fig D - Age-adjusted combined risk factor effects associated with suicide vs other causes of deaths in suicide high-burden states and other Indian states

**.**

**Table A – Annual suicide study deaths, estimated deaths and death rates for India from 2001 to 2019 – both sexes combined**

| **Year** | **Study deaths from Suicides / All causes deaths** | **Proportional Suicide deaths** | **Estimated suicide deaths 000** | **Suicide death rate /100,000** | | | | **Period risk age 15-69 years (%**)† |
| --- | --- | --- | --- | --- | --- | --- | --- | --- |
|  |  |  |  | **All ages ASR** | **15-29 years** | **30-69 years** | **70+ years** |  |
| 2001 | 886 / 41,848 | 2.1% | 179.8 | 16.7 | 30.4 | 20.6 | 23.8 | 1.2 |
| 2002 | 970 / 41,757 | 2.3% | 188.8 | 17.1 | 30.0 | 22.1 | 22.8 | 1.3 |
| 2003 | 883 / 38,834 | 2.3% | 190.0 | 16.8 | 29.4 | 21.9 | 21.5 | 1.3 |
| 2004 | 897 / 37,380 | 2.4% | 202.9 | 17.6 | 30.0 | 23.2 | 24.7 | 1.3 |
| 2005 | 1096 / 46,755 | 2.3% | 204.5 | 17.4 | 30.0 | 22.7 | 24.6 | 1.3 |
| 2006 | 1150 / 47,472 | 2.4% | 209.0 | 17.4 | 29.7 | 22.9 | 25.7 | 1.3 |
| 2007 | 1135 / 48,537 | 2.3% | 202.5 | 16.5 | 28.4 | 21.9 | 22.5 | 1.3 |
| 2008 | 1173 / 47,674 | 2.5% | 203.1 | 16.2 | 27.5 | 21.8 | 22.3 | 1.3 |
| 2009 | 1124 / 47,873 | 2.3% | 202.6 | 15.9 | 27.1 | 21.0 | 25.2 | 1.2 |
| 2010 | 1202 / 45,719 | 2.6% | 211.4 | 16.3 | 27.5 | 21.7 | 24.4 | 1.2 |
| 2011 | 1251 / 46,099 | 2.7% | 218.0 | 16.5 | 27.9 | 22.0 | 23.8 | 1.3 |
| 2012 | 1179 / 46,635 | 2.5% | 218.1 | 16.2 | 27.1 | 21.8 | 23.4 | 1.2 |
| 2013 | 1217 / 45,333 | 2.7% | 217.2 | 15.8 | 25.6 | 21.8 | 23.7 | 1.2 |
| 2014 | 639 / 29,647 | 2.2% | 200.9 | 14.3 | 22.8 | 20.0 | 22.9 | 1.1 |
| 2015 | 984 / 39,592 | 2.5% | 197.4 | 13.8 | 20.8 | 20.4 | 18.3 | 1.1 |
| 2016 | 907 / 39,571 | 2.3% | 181.2 | 12.4 | 18.6 | 18.6 | 17.5 | 1.0 |
| 2017 | 1106 / 46,402 | 2.4% | 189.0 | 12.6 | 18.4 | 19.3 | 20.0 | 1.0 |
| 2018 | 1261 / 48,410 | 2.6% | 194.7 | 12.8 | 18.7 | 19.6 | 18.8 | 1.0 |
| 2019 | 1067 / 43,804 | 2.4% | 201.8 | 13.1 | 19.1 | 19.8 | 20.5 | 1.1 |
| **Overall, 2001-2019 and plausible range (L, U)** | | | | | | | |  |
|  | 20,127 / 829,342 | 2.4% | 3,812.8 | 15.5 | 25.7 | 21.2 | 22.4 | 1.2 |
|  | | (2.1%, 2.7%) | (3790, 3835) | (12, 19) | (17, 34) | (18, 24) | (18, 27) | (1.0, 1.4) |
| Average annual percent change | | | 0.1 | -1.5 | -2.2 | -0.7 | -1.1 |  |

**Notes:**

From 2001-2019 study period 2001-2014 deaths are from Million Death Study and 2015 onwards are from the same study continued by the Registrar General of India. 2015 onwards are approximate and were determined from the three-year rolling death totals published in causes of death reports.^12^ Population estimated by UN-WPP 2022 for India used as the denominator for death rates.^19^ ASR – Age standardized rate. 2001 India population was used as the reference population in age standardization. † Period risk for 15 to 69 years was calculated using the 5 yearly probabilities of death from suicide.

**Table B – Annual suicide study deaths, estimated deaths and death rates for India from 2001 to 2019 - Females**

| **Year** | **Study deaths from Suicides / All causes deaths** | **Proportional Suicide deaths** | **Estimated suicide deaths 000** | **Suicide death rate /100,000** | | | | **Period risk age 15-69 years (%)†** |
| --- | --- | --- | --- | --- | --- | --- | --- | --- |
|  |  |  |  | **All ages ASR ¥** | **15-29 years** | **30-69 years** | **70+ years** |  |
| 2001 | 358 / 18,647 | 1.9% | 76 | 14.6 | 32.6 | 13.9 | 12.6 | 1.0 |
| 2002 | 393 / 18,837 | 2.1% | 79 | 14.9 | 33.1 | 14.0 | 13.4 | 1.0 |
| 2003 | 365 / 17,182 | 2.1% | 80 | 14.7 | 32.2 | 14.2 | 12.0 | 1.0 |
| 2004 | 353 / 16,064 | 2.2% | 87 | 15.6 | 33.9 | 14.9 | 15.3 | 1.1 |
| 2005 | 486 / 20,654 | 2.4% | 91 | 16.0 | 35.2 | 15.1 | 16.2 | 1.1 |
| 2006 | 470 / 20,856 | 2.3% | 93 | 16.1 | 35.9 | 14.6 | 18.3 | 1.1 |
| 2007 | 466 / 21,319 | 2.2% | 88 | 15.0 | 33.1 | 14.2 | 16.0 | 1.0 |
| 2008 | 508 / 20,717 | 2.5% | 87 | 14.5 | 31.5 | 14.1 | 16.0 | 1.0 |
| 2009 | 483 / 21,086 | 2.3% | 87 | 14.2 | 30.9 | 13.8 | 17.3 | 1.0 |
| 2010 | 524 / 19,954 | 2.6% | 92 | 14.9 | 32.3 | 14.1 | 19.8 | 1.0 |
| 2011 | 533 / 19,806 | 2.7% | 95 | 15.0 | 32.5 | 14.4 | 17.8 | 1.0 |
| 2012 | 468 / 20,106 | 2.3% | 93 | 14.6 | 30.7 | 14.2 | 19.4 | 1.0 |
| 2013 | 488 / 19,691 | 2.5% | 91 | 14.0 | 28.4 | 14.6 | 15.9 | 1.0 |
| 2014 | 242 / 11,775 | 2.1% | 84 | 12.7 | 24.7 | 13.6 | 16.5 | 0.9 |
| 2015 | 384 / 15,731 | 2.4% | 83 | 12.3 | 22.6 | 14.9 | 11.1 | 0.9 |
| 2016 | 353 / 15,738 | 2.2% | 77 | 11.1 | 19.9 | 13.7 | 13.5 | 0.8 |
| 2017 | 427 / 19,555 | 2.2% | 79 | 11.1 | 19.5 | 14.1 | 18.3 | 0.8 |
| 2018 | 467 / 20,407 | 2.3% | 80 | 11.1 | 20.0 | 13.8 | 16.5 | 0.8 |
| 2019 | 338 / 18,620 | 1.8% | 79 | 10.9 | 19.7 | 13.3 | 15.6 | 0.8 |
| **Overall, 2001-2019 and plausible range (L, U)** | | | | | | | |  |
|  | 8,106 / 356,746 | 2.3% | 1,621 | 13.9 | 28.9 | 14.2 | 15.9 | 1.0 |
|  | | (1.8%, 2.7%) | (1609, 1633) | (10, 17) | (18, 40) | (13, 15) | (11, 21) | (0.8, 1.1) |
| **Average annual percent change** | | | -0.2 | -1.6 | -2.4 | -0.2 | 0.7 |  |

See Notes for Appendix Table 1 (a)

**Table C – Annual suicide study deaths, estimated deaths and death rates for India from 2001 to 2019 - Males**

| **Year** | **Study deaths from Suicides / All causes deaths** | **Proportional Suicide deaths** | **Estimated suicide deaths 000** | **Suicide death rate /100,000** | | | | **Period risk age 15-69 years (%)†** |
| --- | --- | --- | --- | --- | --- | --- | --- | --- |
|  |  |  |  | **All ages ASR ¥** | **15-29 years** | **30-69 years** | **70+ years** |  |
| 2001 | 528 / 23,201 | 2.3% | 104 | 18.7 | 28.4 | 26.9 | 36.8 | 1.4 |
| 2002 | 577 / 22,920 | 2.5% | 109 | 19.2 | 27.2 | 29.7 | 33.7 | 1.4 |
| 2003 | 518 / 21,652 | 2.4% | 110 | 19.0 | 26.9 | 29.2 | 32.6 | 1.5 |
| 2004 | 544 / 21,316 | 2.6% | 116 | 19.6 | 26.4 | 31.0 | 35.7 | 1.5 |
| 2005 | 610 / 26,101 | 2.3% | 114 | 18.7 | 25.2 | 29.9 | 34.6 | 1.5 |
| 2006 | 680 / 26,616 | 2.6% | 116 | 18.7 | 24.0 | 30.8 | 34.6 | 1.5 |
| 2007 | 669 / 27,218 | 2.5% | 114 | 18.0 | 24.1 | 29.1 | 30.2 | 1.5 |
| 2008 | 665 / 26,957 | 2.5% | 116 | 17.9 | 23.8 | 29.0 | 29.9 | 1.5 |
| 2009 | 641 / 26,787 | 2.4% | 116 | 17.5 | 23.5 | 27.8 | 34.6 | 1.5 |
| 2010 | 678 / 25,765 | 2.6% | 119 | 17.7 | 23.1 | 28.9 | 30.0 | 1.6 |
| 2011 | 718 / 26,293 | 2.7% | 124 | 18.0 | 23.7 | 29.3 | 31.0 | 1.6 |
| 2012 | 711 / 26,529 | 2.7% | 125 | 17.8 | 23.9 | 28.9 | 28.1 | 1.7 |
| 2013 | 729 / 25,642 | 2.8% | 126 | 17.6 | 23.1 | 28.7 | 33.0 | 1.7 |
| 2014 | 397 / 17,872 | 2.2% | 117 | 16.0 | 21.0 | 26.1 | 30.7 | 1.6 |
| 2015 | 600 / 23,861 | 2.5% | 114 | 15.3 | 19.2 | 25.7 | 26.9 | 1.5 |
| 2016 | 554 / 23,833 | 2.3% | 104 | 13.7 | 17.4 | 23.2 | 22.2 | 1.4 |
| 2017 | 679 / 26,847 | 2.5% | 110 | 14.1 | 17.4 | 24.2 | 22.0 | 1.5 |
| 2018 | 794 / 28,003 | 2.8% | 115 | 14.5 | 17.6 | 25.1 | 21.5 | 1.5 |
| 2019 | 729 / 25,183 | 2.9% | 122 | 15.2 | 18.6 | 25.9 | 26.3 | 1.6 |
| **Overall, 2001-2019 and plausible range (L, U)** | | | | | | | |  |
|  | 12,021 / 472,596 | 2.5% | 2,192 | 17.2 | 22.9 | 27.9 | 30.2 | 1.6 |
|  | | (2.2%, 2.9%) | (2180, 2204) | (14, 21) | (16, 30) | (23, 32) | (21, 39) | (1.5, 1.8) |
| **Average annual percent change** | | | 0.3 | -1.5 | -2.0 | -1.0 | -1.9 |  |

See Notes for Appendix Table 1 (a)

**Table D – Percent suicide deaths at home 2001-2014**

| **Classification (% of all suicide deaths)** | **Female** | **Male** | **Both sexes** |
| --- | --- | --- | --- |
| Total suicide deaths in study | 6,138 | 8,667 | 14,805 |
| **Percent died at home by age** | |  |  |
| Less than 15 years *(1.9% )* | 59.1 | 57.3 | 58.3 |
| 15-29 years *(45.3% )* | 61.9 | 56.3 | 59.3 |
| 30-69 years *(48.8% )* | 64.3 | 62.2 | 62.9 |
| Above 70 *(4% )* | 77.3 | 66.4 | 70.2 |
| All ages *(100% )* | 63.2 | 60.2 | 61.5 |
| **Ages 15-69 years** | | |  |
| **Residence** |  |  |  |
| Rural *(83.3% )* | 63.1 | 59.9 | 61.2 |
| Urban *(16.7% )* | 61.5 | 60.1 | 60.7 |
| **Region** | | |  |
| High-burden *(43.8% )* | 64.7 | 60.8 | 62.4 |
| Other states *(56.2% )* | 61.5 | 59.2 | 60.2 |
| **Method of suicide** |  |  |  |
| Poisoning *(40.4% )* | 53.8 | 50.4 | 51.6 |
| Hanging *(37.3% )* | 83.6 | 75.5 | 78.4 |
| Fire *(10.5% )* | 49.1 | 48.4 | 48.9 |
| Other * *(11.8% )* | 56.2 | 45.4 | 50.2 |

Notes: * Other methods include drowning and submersion (ICD 10: X71), firearms (X72-X74) and other methods of suicides (X77-X84). High-burden states are Andra Pradesh, Telangana, Karnataka, Kerala and Tamil Nadu.

**Table E –** **Annual suicide Deaths (in thousands) and death rates per 100,000: Comparison between current study and NCRB data in India, 2001–2019**

|  | **SRS-MDS (Current study)** | | **NCRB** | |
| --- | --- | --- | --- | --- |
| **Year** | **Deaths 000** | **Standardized Death Rate /100,000** | **Deaths 000** | **Standardized Death Rate /100,000** |
| 2001 | 179.8 | 16.7 | 108.5 | 10.1 |
| 2002 | 188.8 | 17.1 | 110.4 | 10.0 |
| 2003 | 190.0 | 16.9 | 110.9 | 9.8 |
| 2004 | 202.9 | 17.6 | 113.7 | 9.8 |
| 2005 | 204.5 | 17.4 | 113.9 | 9.6 |
| 2006 | 209.0 | 17.4 | 118.1 | 9.8 |
| 2007 | 202.5 | 16.5 | 120.7 | 9.9 |
| 2008 | 203.1 | 16.2 | 125.0 | 9.9 |
| 2009 | 202.6 | 15.9 | 127.2 | 10.1 |
| 2010 | 211.4 | 16.3 | 134.6 | 10.3 |
| 2011 | 218.0 | 16.5 | 135.6 | 10.2 |
| 2012 | 218.1 | 16.2 | 120.5 | 10.0 |
| 2013 | 217.2 | 15.8 | 134.8 | 9.7 |
| 2014 | 200.9 | 14.3 | 131.7 | 9.4 |
| 2015 | 197.4 | 13.7 | 133.6 | 9.4 |
| 2016 | 181.2 | 12.4 | 131.0 | 9.1 |
| 2017 | 189.0 | 12.6 | 129.9 | 8.9 |
| 2018 | 194.7 | 12.8 | 134.5 | 9.1 |
| 2019 | 201.8 | 13.1 | 139.1 | 9.3 |
| Overall 2001-2019 | 3,812.8 | 15.5 | 2373.7 | 9.7 |
| Average annual percent change | 0.1% | -1.5% | 1.5% | -0.5% |

Average annual percent change calculated using the linear trend. For both sexes combined analysis, see Appendix Table 1(a). For comparison purposes, both MDS and NCRB death rates were age-standardized to the 2001 Census population. As a result, the NCRB crude death rates published in their annual reports may differ slightly from the standardized rates presented here.

**Table F – Annualized proportional suicide deaths and other mis-classifiable injury, and ill-defined causes of deaths during study periods 2001-5, 2006-10, 2011-14 and 2015-19**

Causes of study deaths from Million Death Study from 2001-2019. Proportional deaths were calculated using cause-specific deaths divided by all causes.

**Table G - Suicide mortality trends from 2001 to 2014 in larger states of India**

| **State** | **Study deaths male/female** | **Estimated total deaths 000 male/female** | **Standardized death rate/100,000 by sex** | | | | | | | |
| --- | --- | --- | --- | --- | --- | --- | --- | --- | --- | --- |
|  |  |  | **Female** | | |  | **Male** | | |  |
|  |  |  | **2001-5** | **2006-10** | **2011-14** | **Average annual rate change%** | **2001-5** | **2006-10** | **2011-14** | **Average annual rate change%** |
| **High-burden southern states** | | | | | | | | | | |
| Andhra Pradesh | 670 / 938 | 153 / 219 | 26.8 | 25.0 | 22.0 | **-2.0** | 38.2 | 33.1 | 30.3 | **-2.3** |
| Tamil Nadu | 979 / 1105 | 166 / 185 | 30.0 | 32.7 | 29.7 | **0.0** | 35.8 | 31.2 | 30.0 | **-1.7** |
| Kerala | 381 / 879 | 42 / 86 | 16.9 | 15.5 | 13.4 | **-2.3** | 33.0 | 30.5 | 28.6 | **-1.4** |
| Karnataka | 617 / 1006 | 105 / 155 | 21.1 | 25.4 | 24.8 | **1.9** | 28.9 | 36.2 | 33.9 | **2.0** |
| **All high-burden** | **2647 / 3928** | **466 / 644** | **25.2** | **26.1** | **23.9** | **-0.5** | **34.7** | **33.0** | **31.0** | **-1.1** |
| **Other states** | | | | | | | | | | |
| Odisha | 263 / 256 | 45 / 45 | 18.8 | 14.3 | 12.9 | **-3.5** | 18.6 | 13.2 | 11.5 | **-4.4** |
| Assam | 116 / 161 | 24 / 36 | 13.9 | 14.8 | 10.5 | **-2.3** | 20.6 | 15.8 | 13.5 | **-3.9** |
| Haryana | 122 / 235 | 18 / 34 | 15.3 | 12.0 | 9.6 | **-4.2** | 22.3 | 17.5 | 16.0 | **-3.1** |
| Jammu & Kashmir | 54 / 56 | 6 / 6 | 7.2 | 8.0 | 9.7 | **3.5** | 8.7 | 6.8 | 7.0 | **-2.0** |
| Chhattisgarh | 86 / 152 | 20 / 46 | 15.7 | 11.4 | 10.4 | **-3.8** | 28.8 | 27.8 | 23.5 | **-2.0** |
| Maharashtra | 298 / 543 | 98 / 166 | 13.8 | 12.1 | 11.8 | **-1.6** | 20.8 | 18.2 | 17.4 | **-1.8** |
| Bihar | 83 / 68 | 37 / 31 | 8.3 | 6.5 | 5.1 | **-4.5** | 4.9 | 5.3 | 4.2 | **-1.4** |
| West Bengal | 688 / 734 | 119 / 128 | 19.6 | 18.6 | 16.3 | **-1.9** | 19.2 | 17.9 | 16.6 | **-1.4** |
| Delhi | 38 / 69 | 7 / 12 | 7.7 | 6.2 | 5.7 | **-2.9** | 11.6 | 10.1 | 10.2 | **-1.3** |
| Madhya Pradesh | 232 / 321 | 70 / 98 | 17.7 | 14.9 | 14.1 | **-2.2** | 19.0 | 19.5 | 19.0 | **0.0** |
| Punjab | 63 / 137 | 11 / 20 | 3.8 | 6.6 | 7.0 | **8.8** | 9.5 | 9.6 | 9.6 | **0.1** |
| Gujarat | 263 / 316 | 59 / 69 | 11.9 | 15.7 | 15.9 | **3.6** | 15.0 | 15.5 | 15.8 | **0.5** |
| Jharkhand | 32 / 50 | 15 / 22 | 8.4 | 6.3 | 9.4 | **2.6** | 13.1 | 13.7 | 13.9 | **0.6** |
| Uttar Pradesh | 333 / 365 | 147 / 167 | 10.9 | 12.0 | 13.5 | **2.4** | 11.5 | 12.1 | 13.6 | **1.9** |
| Rajasthan | 149 / 224 | 37 / 53 | 10.4 | 7.9 | 7.9 | **-2.5** | 11.0 | 10.9 | 13.5 | **2.5** |
| **All other** | **2820 / 3687** | **713 / 934** | **13.4** | **12.5** | **12.4** | **-0.8** | **15.6** | **14.6** | **14.3** | **-0.9** |
|  |  |  |  |  |  |  |  |  |  |  |
| **All India** | **6137 / 8665** | **1223 / 1626** | **15.2** | **14.9** | **14.1** | **-0.8** | **19.0** | **18.0** | **17.4** | **-0.9** |

**Notes** Larger states are those with population >10 million. High-burden states (where suicide mortality rate exceeds 20 per 100,000 population are Andhra Pradesh (includes Telangana), Tamil Nadu, Kerala, Karnataka. We used 2001 population as the reference population for age-adjusted death rates. States are in ascending order of the average annual rate change of age adjusted death rate/100,000 of males. The sub-national analysis is confined to 2001-2014.

| **Female** | **Male** |
| --- | --- |
| **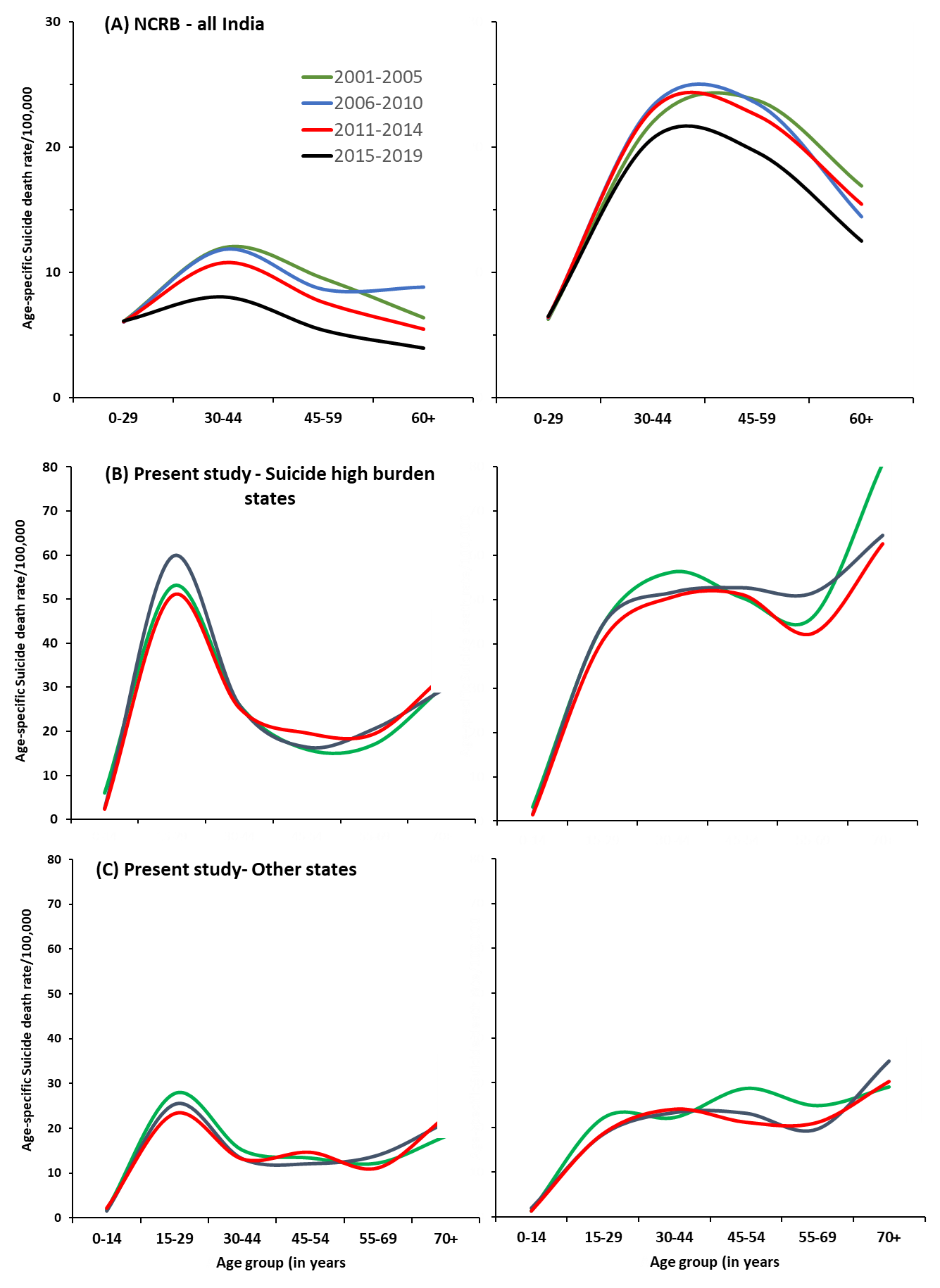** | |

**Fig A** **– Age-sex patterns of suicide deaths observed in NCRB (All-India) and in the present study across High-burden and Other states**

Panels: (A) NCRB – All India, (B) Present Study – Suicide High-Burden States, (C) Present Study – Other States

High-burden states include Andhra Pradesh/Telangana, Karnataka, Kerala, Tamil Nadu. Other states are remaining states. Each curve represents annual average age specific death rates calculated for periods 2001-5, 2006-10, 2011-14 and 2015-19. The X-axis age grouping of present study are slightly different from NCRB because NCRB uses different age groups. The scale of the Y-axis in (A) is lower than that for (B) and (C), given annual death rates by NCRB are under-estimates.

**
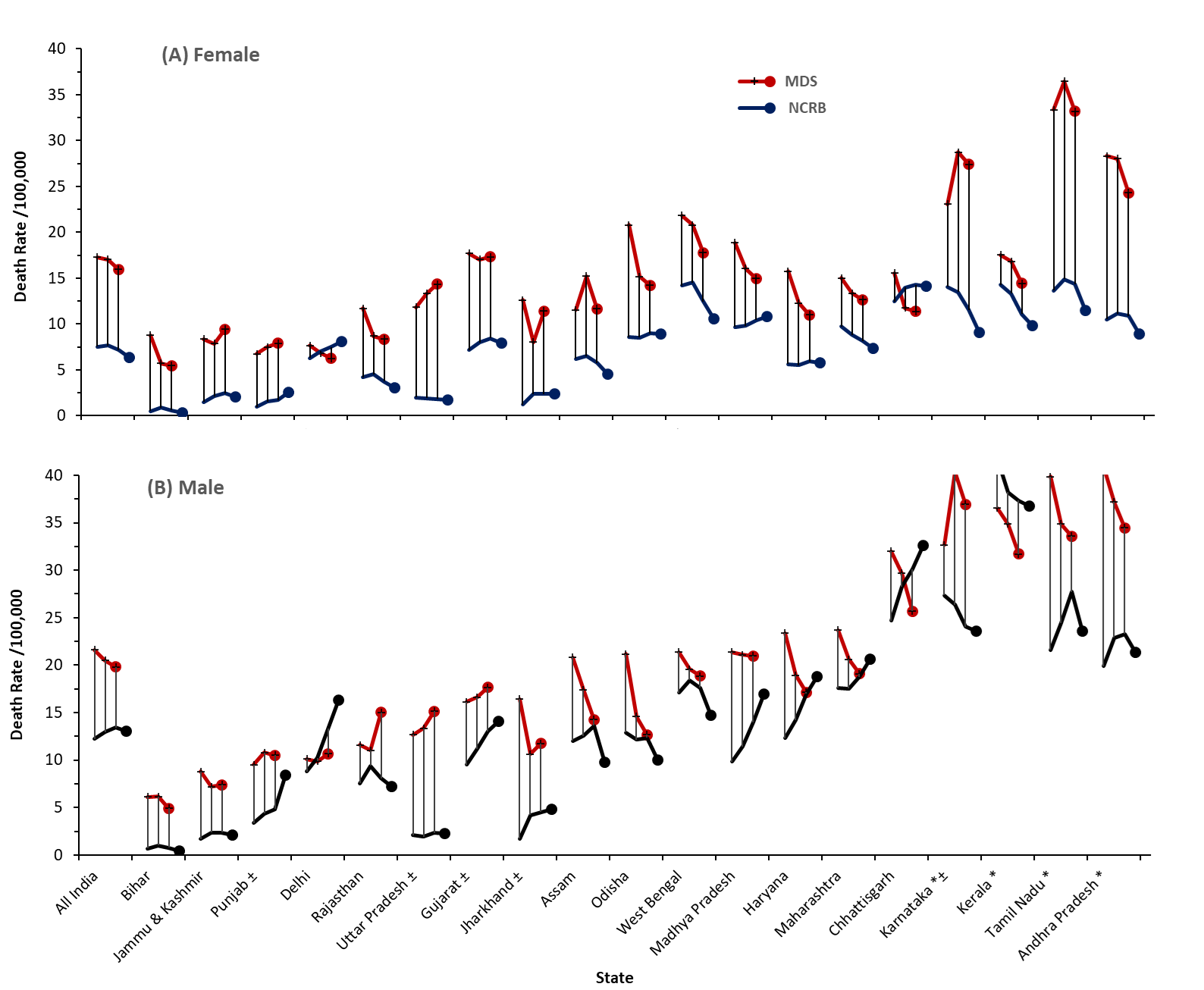
**

**Fig B - State-wise comparison of suicide mortality trends for 2001–2019 between the present study (MDS) and the NCRB**

Panels: (A) Females, (B) Males

The Pearson correlation coefficients (95% CI) between MDS and NCRB for females were 0.73 (0.57, 0.83), and for males were 0.85 (0.76, 0.91). Crude death rates per 100,000 population for all age groups were analyzed. Due to fluctuating annual death rates, we utilized periodic death rates calculated for the periods 2001-5, 2006-10, 2011-14, and 2015-19 in constructing the curves. Except for NCRB, 2015-19 state-wise death rates were not available in MDS. The states on the X-axis are arranged in ascending order of MDS male death rates observed for the baseline period 2001-5.

| 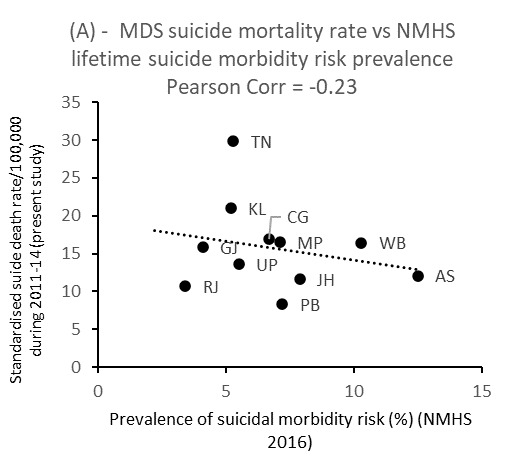 | 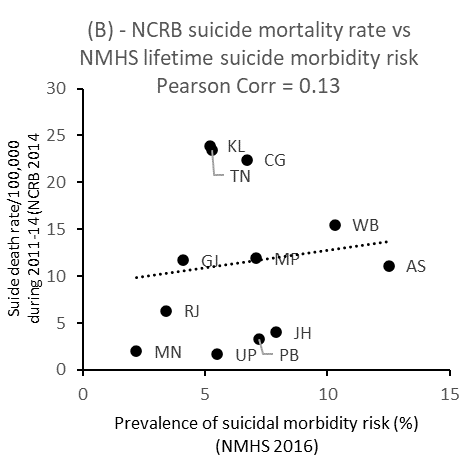 |
| --- | --- |
| 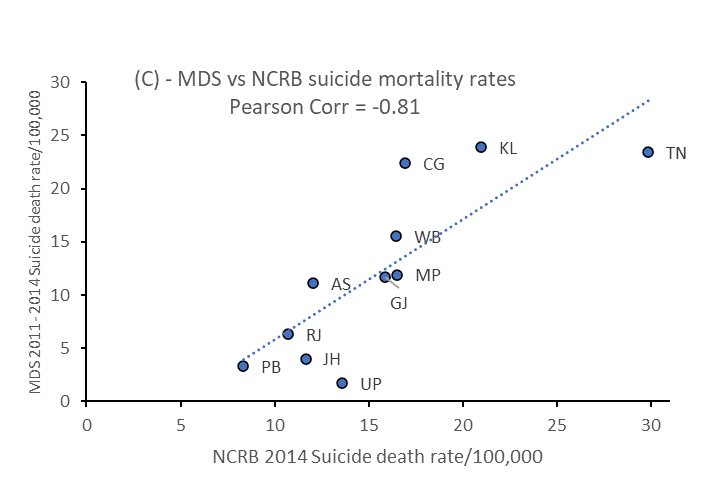 | |
| Abbreviations used for states: Assam (﻿AS), Chhattisgarh (CG), Gujarat (GJ), Jharkhand (JH), Kerala (KL), Madhya Pradesh (MP), Manipur (MN), Panjab (PB), Rajasthan (RJ), Tamil Nadu (TN), Uttar Pradesh (UP), and West Bengal (WB**).** | |

**Fig C – Correlation between prevalence of suicide morbidity risk in states surveyed by National Mental Health Survey (NMHS) 2016 and suicide mortality rates from the present study and NCRB data**

Suicidal morbidity risk was calculated using the information of 4,802 person surveyed in 12 states namely Punjab, Uttar Pradesh, Tamil Nadu, Kerala, Jharkhand, West Bengal, Rajasthan, Gujarat, Madhya Pradesh, Chhattisgarh, Assam and Manipur in NMHS survey 2015-16.^15^ Panels (A) and (B) investigate the associations between prevalence of suicidal morbidity risk (lifetime suicidal morbidity risk (%) estimated in NMHS for 2015-16 period) and the mortality rates estimated for 2011-14 from present study and suicide death rates from the NCRB. Panel (C) illustrates the correlation of death rates between and comparison between present study and NCRB. Pearson correlation coefficient was used to investigate the association between two variables in each plot.

| **High-burden southern states** | **Other states** |
| --- | --- |
|  |  |
|  | |

**Fig D – Age-adjusted combined risk factor effects associated with suicide vs other causes of deaths in suicide high-burden states and other Indian states**

Age adjusted Mantel-Hanzal odds ratios (OR) and the Wald’s 95% confidence intervals examine suicides compared to all other causes of death. The combined risk factors represent the risk of suicide among individuals exposed to each combination of risk factors vs. corresponding reference categories. The analysis examined 472,000 verbal autopsy causes of deaths of age over 15 years from 2001 to 2014. Reference groups are urban for residence; any family member aside from son or daughter in law for relationship to head of household; no- drinker for alcohol use.
